# The Effect of an Aerobic Exercise Programme on Blood Glucose Level, Cardiovascular Parameters, Peripheral Oxygen Saturation, and Body Mass Index among Southern Nigerians with Type 2 Diabetes Mellitus, Undergoing Concurrent Sulfonylurea and Metformin Treatment

**DOI:** 10.21315/mjms2019.26.5.8

**Published:** 2019-11-04

**Authors:** Charles Ikechukwu Ezema, Emmanuel Omeh, Ogochukwu Kelechi Kizito Onyeso, Canice Chukwudi Anyachukwu, Maduabuchukwu Joseph Nwankwo, Augustine Amaeze, Josephine Ugochi Ugwulebor, Emmanuel Okechukwu Nna, Edwin Obiora Ohotu, Ikechukwu Ugwuanyi

**Affiliations:** 1 Department of Medical Rehabilitation, Faculty of Health Sciences and Technology, College of Medicine, University of Nigeria, Enugu, Nigeria; 2 Department of Medical Rehabilitation, Faculty of Health Sciences and Technology, College of Health Sciences, Nnamdi Azikiwe University, Anambra, Nigeria; 3 Safety Molecular Pathology Laboratory, Ranger’s Avenue, Enugu, Nigeria; 4 Department of Haematology and Immunology, University of Nigeria Teaching Hospital, Ituku-Ozalla. Enugu, Nigeria

**Keywords:** aerobic exercise, body mass index, cardiovascular parameters, peripheral oxygen saturation, type-2 diabetes mellitus

## Abstract

**Background:**

Diabetes mellitus increases the risk of cardiovascular diseases and all-cause mortality. The present study investigated the effect of an eight-week aerobics programme on fasting blood sugar (FBS), cardiovascular parameters, peripheral oxygen saturation (SpO_2_), and body mass index (BMI) among subjects with type-2 diabetes mellitus (T2DM).

**Methods:**

A pretest-posttest experimental design was employed. Fifty subjects, diagnosed with T2DM, attending the Diabetes Clinic of the University of Nigeria Teaching Hospital, Enugu, were conveniently recruited, gender and age-matched, and randomised into exercise and control groups. The intervention included an eight-week aerobic exercise at 60%–79% HRmax for 45 min–60 min, 3-days per week. The FBS, SpO_2_, BMI, resting heart rate (RHR), and systolic (SBP) and diastolic blood pressure (DBP) of the subjects were measured before and after the intervention. The paired and independent *t*-test(s) were used for the analyses within and between the groups, respectively (*P* ≤ 0.05).

**Results:**

The exercise group had a significantly lower SBP (15.0 mmHg, *P* = 0.001), DBP (7.9 mmHg, *P* = 0.001), RHR (4.8 bpm, *P* = 0.001), FBS (34.9 mg/dl, *P* = 0.001), and BMI (2.3, *P* = 0.001), while the SpO_2_ improved by 3.9% with *P* = 0.001, relative to the control group.

**Conclusion:**

Aerobics is an efficacious adjunct therapy in controlling the FBS level, blood pressure, BMI, and improving SpO_2_ among T2DM subjects.

## Introduction

Diabetes mellitus (DM) is a group of metabolic diseases characterised by hyperglycaemia resulting from defects in insulin secretion, insulin action or both (1). The aetiology of type-2 diabetes mellitus (T2DM) is not well understood, though a multifactorial model suggested that it arises from the combination of genetic predisposition and environmental factors (2). Important risk factors include a family history of diabetes, increased age, hypertension, lack of physical exercise, obesity, and ethnic background (2, 3). DM is reported worldwide, but research has predicted that by 2030, Asia and Africa will have the highest prevalence of DM due to increasing urbanisation, ‘Western-style’ diet and lifestyle modification (4).

Treatment of diabetes involves a combination of exercise, diet, medication, and daily self-care (5). The first line of treatment for T2DM is self-care, exercise, and diet. Metformin medication is prescribed if the blood sugar is not controlled as desired (6). Subsequently, sulfonylurea can be introduced as a single medication or in combination with metformin. There are concerns about increased risks of obesity for sulfonylureas (7) and cardiovascular mortality for both metformin and sulfonylureas (8).

Therefore, T2DM and its oral medications increase the risk of all-cause mortality and cardiovascular diseases (CVD) (9). Hence, the American Diabetes Association (ADA) recommended that individuals with T2DM should perform at least 150 min of moderate-intensity aerobic exercise or at least 90 min of vigorous aerobic exercise per week, due to the proven benefits of aerobic exercises in T2DM (10).

Most of the studies that investigated diabetes and associated factors were conducted using Whites, Caucasians and other races, except pure black Africans. However, studies have shown interracial, interpersonal, and ethnic variation in susceptibility to T2DM (3, 11–13). Therefore, the purpose of the present study was to investigate the effect of an adjunct eight-week exercise programme on fasting blood sugar (FBS), cardiovascular parameters, body mass index (BMI) and peripheral oxygen saturation (SpO_2_) among pure Black African subjects with T2DM, currently on oral sulfonylurea and metformin combined therapy.

## Methods and Subjects

### Study Design

The study employed an age and gender-matched randomised controlled pretest-posttest experimental design.

### Participants

**Figure 1:**
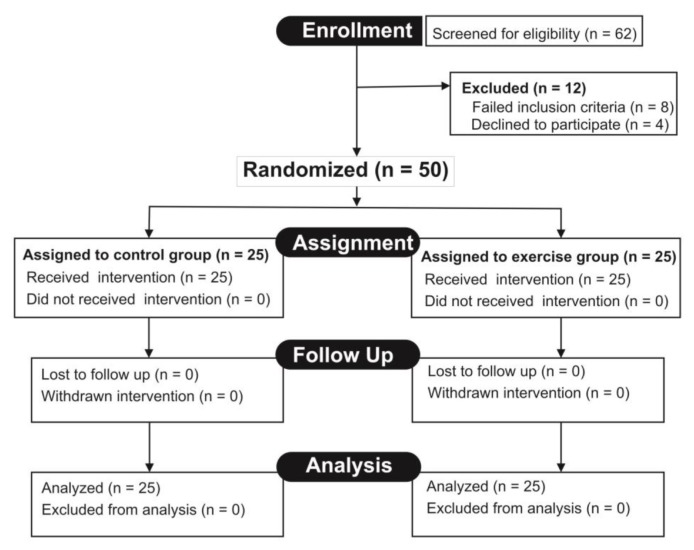
Trial profile

A sample size of 54 was calculated at a 95% power and 0.05 level of error with an effect size of 0.5 using G* power 3.0.10 software. Sixty-two patients were conveniently recruited from the Diabetes Clinic of University of Nigeria Teaching Hospital (UNTH) and screened with the selection criteria. Eight patients were excluded, while four patients withdrew. Fifty patients that meet the inclusion criteria (22 males and 28 females, aged between 42 and 63 years) volunteered to participate in the study and gave their individual informed consent in accordance with the American College of Sports Medicine guidelines (14). An independent assessor arranged the subjects according to gender in ascending order of age and assigned them alternatively into exercise and control groups (Figure 1).

### Selection Criteria

Inclusion of subjects was based on the following criteria: T2DM patient of UNTH, medically stable, without any cardiac complications, blood pressure (BP) between 90/60 mmHg to 140/90 mmHg, on diet and/or oral drugs only, sedentary prior to the study, no psychiatric or psychological disorders and passed the Young Men Christian Association’s sub-maximal cycle ergometry stress test protocol for cardiovascular fitness (15, 16).

Exclusion criteria were as follows: subjects with uncontrolled hyperglycaemia (> 250 mg/dL), retinopathy, vascular, cardiac, renal, or respiratory diseases; currently on insulin therapy, vigorous physical activities, alcohol use, smoker, obese (BMI > 30 kg/m^2^) or underweight (BMI < 20 kg/m^2^).

### Measurements

Prior to the training programme, the baseline characteristics of the subjects were measured using a BMI apparatus (RGZ-120; made in China) (weight [kg]/ height [m]^2^ = BMI). The resting heart rate (RHR), SBP and DBP were monitored using an automated electronic device [Omron digital BP monitor, Model 11 EM 403c; Tokyo Japan] (17). The SpO_2_ was measured by applying the pulse oximeter (MQ3200; made in China) on the patients’ thumb after 10 min rest in supine lying and the fasting plasma glucose level (FBS) was tested using a glucometer (Accu Check, Active; made in Germany).

The FBS values were taken not later than 9:00 a.m. and at least 12 h post anti-diabetes medication. The blood sample was taken through the cubital-vein and used immediately. All the parameters were measured thrice, and the median values recorded. The parameters were re-measured for both groups, one-day after the eight weeks of the intervention.

### Intervention Procedures

The patients were advised to maintain a balanced diabetes exchange diet that provides approximately 50%–60% of calories from carbohydrates, 10%–20% from protein, and < 30% from fat. Each patient was on oral therapeutic dosages of metformin and sulfonylureas (glibenclamide, glimepiride or glipizide) as prescribed by the physician.

The exercise group was engaged in a continuous aerobic programme for eight weeks at the gymnasium in the Department of Medical Rehabilitation, University of Nigeria, Enugu Campus. The researchers employed a moderate intensity (60%–79% of a subject’s HRmax) bicycle ergometer (18, 19).

First, the subjects were allowed to get acquainted with the programme by warming-up by pedalling at 50 rpm and zero workload for 10 min. Then, the bicycle workload was set at 100 kgm/min (17 watts) and was increased gradually until an intensity of 60% of the HRmax was obtained and maintained for 45 min. The intensity was progressed to 79% HRmax for 60 min within the first two weeks and was maintained till the eighth week.

Each exercise session ended with a 10 min cool-down by pedalling at zero resistance. While exercising, the subjects were monitored for their perceived exertion rate on the Borg’s scale. They were also instructed to report any symptom that would necessitate the termination of the exercise.

All the subjects in the exercise group completed the programme thrice per week for the required eight weeks, uneventfully. The gymnasium opened between 8:00 a.m. and 4:00 p.m. on weekdays and 8:00 a.m. and 10:00 a.m. on Saturdays. The patients were scheduled for three alternate days per week, based on convenience.

The subjects in the control group were instructed not to undertake any organised or structured physical activity apart from the activities of daily living during the eight-week period of study.

The subjects had access to self-test through personal glucometer at home or in their neighbourhood. In case of any hypoglycaemic event, the subjects were advised to take a bottle of sugar-containing drink and report to the nearest clinic.

### StatisticalAnalysis

The data collected were analysed with SPSS, version 20 software. Chi-square test and independent *t*-test were used to analyse the sex and age distribution, respectively, between the groups. The paired samples and independent *t*-test(s) were used to compare the mean (SD) values of the parameters, pre- and post-intervention, within and between the groups, respectively. Values of *P* ≤ 0.05 were considered statistically significant.

## Results

The CONSORT flowchart (Figure 1) shows that 50 subjects were randomly and equally assigned to the exercise and control groups (*n* = 25 each). The subjects concluded the study and were included in the analysis. The study was age- and gender-matched. Each group comprised of 14 women and 11 men, and there were no significant differences in gender distribution across the groups (*χ*^2^ = 0.000, *P* = 1.000). The baseline subject characteristics (Table 1) show that there was no significant difference between the mean (SD) age of the exercise group [51.7 (5.0) years] and the control group [51.5 (5.0) years, *t* = 0.226, *P* = 0.822]. Following the interventions, the FBS of the subjects in the exercise group reduced relative to the control group (Table 2), the mean difference (MD) was 34.8 mg/dl (95% CI: 26.8 mg/dl, 42.8 mg/dl), *t* = 8.752, *P* = 0.001.

There were changes in the cardiovascular parameter of the subjects in both groups (Table 3), which resulted in a significant decrease in SBP of the subjects in the exercise group relative to the control group; the MD was 15.0 mmHg (95% CI: 12.2 mmHg, 18.0 mmHg, *t* = 10.333, *P* = 0.001). The DBP of subjects in the exercise group declined significantly compared with the control group; the MD was 7.9 mmHg (95% CI: 5.8 mmHg, 10.0 mmHg, *t* = 7.558, *P* = 0.001). Similarly, the exercise group had significantly decreased RHR; the MD was 4.8 bpm (95% CI: −1.3 bpm, 4.1 bpm, *t* = 3.792, *P* = 0.001), relative to the control group.

Likewise, the subjects in the exercise group had a significantly better peripheral oxygen saturation relative to those in the control group; SpO_2_ difference was −3.9% (95% CI: −5.6%, −2.3%, *t* = −4.812, *P* = 0.001). Table 4 shows that subjects in the exercise group lost some weight during the programme, which led to a significant decrease in their BMI, with the MD of 2.3 (95% CI: 1.8, 2.9, *t* = 8.687, *P* = 0.001), as compared with those in the control group.

### Harm

It was noteworthy that three subjects (one from the exercise group and two from the control group) reported a hypoglycaemic event in at least one day that they skipped one or two meals during the period of this study. However, there was no report of any hyperglycaemic crisis or any other harm associated with this study.

## Discussion

T2DM and its oral medications are associated with increased risk of CVD and all-cause mortality (9). The findings from this study have shown a significant decrease in FBS, SBP, DBP, RHR, and BMI and an increase in SpO_2_ in the exercise group when compared with the control group. The desired outcome in the blood glucose level, cardiovascular parameters, oxygen saturation, and body mass of patients who participated in the exercise programme is consistent with those reported in a few other studies (19, 20).

Following the interventions, subjects in both the exercise and control groups had a significantly reduced FBS since both groups were under oral hypoglycaemic agents (OHA). However, comparison of the post-intervention outcomes showed that the exercise group had a significantly lower FBS than the control group. This observation strengthens the role of exercise as an efficacious adjunct therapy in the management of people with T2DM. A randomised control study (*n* = 60) investigated the effect of 6-month aerobic exercise training (four times/week, 45–60 min/session) on glucose control in T2DMand overweight individuals on oral antidiabetic regimen. Another study (*n* = 77) demonstrated that six months of Hatha yoga and regular aerobic exercise decreased FBS in patients with T2MD by 29.48% and 27.43%, respectively (22).

Diabetes and OHA have been reported as important risk factors in the development of CVD (7–9), but aerobic exercises lower the risk (10). Kadoglou et al. (21) reported that in comparison with the baseline and control group, exercise-treated patients improved glucose control, exercise capacity (VO_2_ peak), and exhibited decreased insulin resistance and SBP considerably. Similarly, the present study showed that subjects in the exercise group had decreased SBP, DBP, and RHR compared to their counterparts in the control group.

The mechanism by which regular aerobic training reduces blood glucose level could be linked to three major pathways: the acute stimulation of muscle glucose transport pathway, acute enhancement of insulin action, and long-term up-regulation of the insulin signalling pathway (23–25). Wilmore and Costil (26) postulated that the reduction in FBS in the exercise group occurs because aerobic bouts of exercise training enhance anaerobic and aerobic energy systems of the motor units, leading to more efficient utilisation of fats and carbohydrates. The merit of exercise training for T2DM patients is not limited to its antihyperglycemic effect; it offers the opportunity for reduction of therapeutic doses of medications needed to achieve euglycemia in the subjects (6). Bell (27) posited that cardiovascular risk factors of oral antidiabetic agents are dose dependent.

There is no gainsaying that exercise modifies the sequelae of T2DM, which includes obesity and CVD (2). Although it has been reported that oral sulfonylureas increase the risk of obesity among users (8), the present study has shown (Table 4) that the subjects in the control group gained weight, while their counterparts, who participated in exercise training, had a significantly reduced BMI. Cheng and Kashyap (28) opined that both diabetes and obesity are associated with an increase in morbidity and mortality from CVD. They suggested that treatment of diabetes should not focus on glycaemic control as its sole intention, but it should focus on the effect of those medications on weight. This study has shown that aerobics can ameliorate the side effects of drug induced-obesity among T2DM patients on OHA.

Garg et al. (29) reported that obesity is a strong independent contributor to the reduction in SpO_2_ in ambulatory T2MD patients. Tissue hypoxia is an important contributor to diabetes complications. The result of this study has shown significant improvement (3.9%) in SpO_2_ of subjects in the exercise group. Prescribed exercises are known to decrease all-cause morbidity resulting from cardiopulmonary and vascular risk factors. There is an association between obesity, glycation of haemoglobin, and SpO_2_ among T2DM patients (29). Hence, this study hypothesises that exercise can reduce chronic hypoxia-related micro- and macro-vascular changes in tissues and organs of T2DM patients by improving circulation at the extremities.

Prior to the present study, the researchers did not know of any other work that demonstrated an adjunct role of continuous aerobic exercise training in the normalisation of blood glucose, cardiovascular parameters, and improvement of SpO_2_ among pure black subjects undergoing combined oral metformin and sulfonylurea medications for T2DM.

However, limitations of the study include short duration (eight weeks) mono-exercise regimen (same frequency, intensity, and mode), inability to analyse the glycated haemoglobin (HbA1c) level of the subjects, and lack of follow-up. Some overweight-hyperglycaemic subjects were used for the study. It implies that the data from the pulse oximetry (SpO_2_) should be interpreted with caution, following the report that elevated HbA1c levels lead to overestimation of arterial oxygen saturation (SaO_2_) by pulse oximetry (28).

Although the exercise group had remarkably higher FBS and SBP relative to the control group at the baseline, this observation could be attributed to a randomisation error. The findings of the study showed a significant post-intervention improvement in FBS and SBP of the exercise group, compared with the control group. In spite of the limitations, the findings of this study deserve attention in future studies.

## Conclusion

The eight-week aerobic exercise programme was found to be an effective adjunct therapy in controlling blood glucose level, blood pressure, BMI, and improving SpO_2_ among T2DM subjects. Therefore, we recommend that clinicians should prescribe continuous aerobic exercise training as a non-pharmacological adjunct for the prevention and management of T2DM and its sequelae.

## Figures and Tables

**Table 1:** Baseline subject characteristics (*n* = 50)

		Exercise group (*n* =25) *n (%)*	Control group (*n* = 25) *n (%)*	Total *n (%)*
Gender: b	Female	14 (56.0)	14 (56.0)	28 (56.0)
	Male	11 (44.0)	11 (44.0)	22 (44.0)
Age (years)^a^, ^c^		51.7 (5.0)	51.4 (5.0)	51.5 (5.0)
BMI (kg/m^2^) a		25.6 (3.1)	25.4 (3.0)	25.5 (3.0)
FBS (mg/dL) a		175.3 (17.2)	165.0 (18.0)	170.1 (18.2)
SBP (mmHg) a		131.9 (6.4)	127.3 (8.1)	129.6 (7.6)
DBP (mmHg) a		79.3 (2.8)	77.3 (4.6)	78.3 (3.9)
RHR (bpm) a		76.5 (4.4)	75.1 (4.9)	75.8 (4.7)
SpO_2_ (%)^a^		95.9 (2.1)	95.2 (3.5)	95.5 (2.9)

BMI = body mass index, FBS = fasting blood sugar, SBP = systolic blood pressure

DBP = diastolic blood pressure, RHR = resting heart rate, SpO_2_ = peripheral oxygen saturation

a mean (SD),

b Gender, *χ*^2^-statistic (df) = 0.000 (1), *P* =1.000,

c Age, *t*-statistic (df) = 0.226 (48), *P* = 0.822

No significant difference in gender or age distribution between the two groups ( *P* < 0.05)

**Table 2:** Comparison of FBS pre- and post-intervention for exercise and control groups

Variables	Mean (SD)	Mean (SD)	Mean difference (95% CI)	*t*-statistic (df)	*P*-value
FBS level (mg/dl)	Pre-trial	Post-trial			
Exercise group (*n* = 25)	175.3 (17.2)	124.0 (7.7)	51.3 (44.9, 57.6)	16.67 (24) a	0.001^c^
Control group (*n* = 25)	165.0 (18.0)	148.6 (15.2)	16.4 (11.2, 21,6)	6.48 (24) a	0.001^c^
Mean difference (95% CI**)**	10.3 (0.3, 20.1)	−24.6 (−31.4, −17.1)	**34.9 (26.8, 42.8)**	**8.75 (48)** b	**0.001**^c^

FBS = fasting blood sugar,

a Paired *t*-test,

b Independent *t*-test,

c *P*-value is significant at *P* < 0.05

**Table 3:** Comparison of cardiovascular parameters pre- and post-intervention for exercise and control groups

Variables	Mean (SD)	Mean (SD)	Mean difference (95% CI)	*t*-statistic (df)	*P*-value
	Pre-trial	Post-trial			
Exercise group SBP (mmHg)	131.9 (6.4)	119.2 (6.1)	12.7 (10.3, 15.2)	10.77 (24) a	0.001 c
Control group SBP (mmHg)	127.3 (8.1)	129.6 (8.1)	−2.3 (−4.1, −0.6)	−2.72 (24) a	0.012 c
Mean difference (95% CI)	4.6 (0.5, 8.8)	−10.4 (−14.5, −6.4)	**15.0 (2.2, 18.0)**	**10.33 (48)** b	**0.001** c
Exercise group DBP (mmHg)	79.3 (2.8)	71.7 (4.2)	7.6 (5.8, 9.4)	8.73 (24) a	0.001 c
Control group DBP (mmHg)	77.3 (4.6)	77.6 (5.0)	−0.3 (−1.5, 0.9)	−0.49 (24) a	0.631
Mean difference (95% CI)	2.0 (−0.2, 4.2)	5.9 (−8.6, −3.3)	**7.9 (5.8, 10.0)**	**7.56 (48)** b	**0.001** c
Exercise group RHR (bpm)	76.5 (4.4)	72.8 (3.6)	3.7 (2.9, 4.5)	9.66 (24) a	0.001 c
Control group RHR (bpm)	75.1 (4.9)	74.0 (4.1)	1.1 (−0.1, 2.3)	1.86 (24) a	0.075
Mean difference (95% CI)	1.4 (−1.3, 4.1)	−1.2 (−3.4, 0.9)	**4.8 (**−**1.3, 4.1)**	**3.79 (48)** b	**0.001** c

SBP = systolic blood pressure, DBP = diastolic blood pressure, RHR = resting heart rate

a Paired *t*-test,

b Independent *t*-test,

c *P*-value is significant at *P* < 0.05

**Table 4:** Comparison of oxygen saturation, and BMI pre- and post-intervention for exercise and control groups

Variables	Mean (SD)	Mean (SD)	Mean difference (95% CI)	*t*-statistic (df)	*P*-value
	Pre-trial	Post-trial			
Exercise group SpO_2_ (%)	95.9 (2.1)	99.3 (0.9)	−3.4 (−4.2, −2.6)	−0.86 (24) a	0.001 c
Control group SpO_2_ (%)	95.2 (3.5)	94.7 (2.5)	0.5 (−0.9, 2.0)	0.73 (24) a	0.472
Mean difference	0.7 (−1.0, 2.3)	4.6 (3.5, 5.7)	−**3.9 (**−**5.6**, −**2.3)**	−**4.812 (48)** b	**0.001** c
Exercise group BMI	25.61 (3.1)	23.92 (3.0)	1.7 (1.2, 2.1)	7.74 (24) a	0.001 c
Control group BMI	25.42 (3.0)	26.06 (2.9)	−0.6 (−1.0, −0.3)	−4.11 (24) a	0.001 c
Mean difference	0.2 (−1.5, 1.9)	−2.1 (−3.8, −0.4)	**2.3 (1.8, 2.9)**	**8.69 (48)** b	**0.001** c

SpO_2_ = peripheral oxygen saturation, BMI = body mass index,

a Paired *t*-test,

b Independent *t*-test,

c *P*-value is significant at *P* < 0.05
